# The reproductive outcome of an infertile man with AZFc microdeletions, via intracytoplasmic sperm injection in a high-risk pregnancy: Case report and literature review

**DOI:** 10.1097/MD.0000000000016358

**Published:** 2019-07-12

**Authors:** Cong Hu, Xiangyin Liu, Linlin Li, Xiaonan Hu, Haibo Zhu, Dongfeng Geng, Ruizhi Liu, Ruixue Wang

**Affiliations:** Center for Reproductive Medicine, Center of Prenatal Diagnosis, First Hospital, Jilin University, Changchun, China.

**Keywords:** AZFc microdeletions, ICSI, Inv (9), male infertility, prenatal diagnosis

## Abstract

**Rationale:**

Infertile men with Y-chromosome microdeletions have been reported to be able to have their own children via intracytoplasmic sperm injection (ICSI).

**Patient concerns:**

A 27-year-old man with Y-chromosome azoospermia factor c (AZFc) deletions underwent ICSI treatment. The pregnancy showed a high risk for trisomy 21 syndrome (risk value: 1 in 150).

**Diagnoses:**

The karyotype of the patient was 46, XY, inv (9) (p11q13). His wife had a normal karyotype. Sequence-tagged site-based polymerase chain reaction (PCR) analysis showed that markers sY254 and sY255 were absent. ICSI was performed. Two embryos (6IV, 8II) were transferred to the uterus of the patient's wife. Second-trimester maternal serum triple-screening showed that the pregnancy was high risk for trisomy 21 syndrome (risk value: 1 in 150). Amniocentesis was performed and revealed that the fetal chromosomal karyotype was 46, XX, inv (9) (p11q13).

**Interventions:**

The couple chose to continue the pregnancy and a healthy girl was born at 39 weeks of gestation.

**Outcomes:**

An infertile man with AZFc microdeletions can reproduce via ICSI technology. The karyotype inv (9) (p11q13) can be transmitted to offspring. Whether this karyotype has clinical significance, such as causing infertility or variations in prenatal biochemical markers, is unclear.

**Lessons:**

Y-chromosome microdeletions and/or the karyotype inv (9) (p11q13) may cause clinically significant variation in prenatal biochemical markers.

## 1 Introduction

Infertility affects around 1 in 6 couples of reproductive age and in approximately half of these cases infertility is due to male factors. After Klinefelter syndrome (KS or 47, XXY), Y-chromosome microdeletions are the most well-known genetic causes of male infertility.^[1]^

The azoospermia factor (AZF) locus in Yq11 contains the genes necessary for normal spermatogenesis, and deletions in this locus have been correlated with male infertility.^[2]^ The AZF region comprises three subregions: AZFa, AZFb, and AZFc. It is generally assumed that the identification of sperm in men with AZFa or AZFb region deletions is unlikely, whereas men with AZFc deletions have a higher chance (up to 70%) of having sperm identified upon surgical sperm extraction.^[3]^ However, the functional contribution to spermatogenesis of genes from each subregion has not been rigorously investigated, and the findings of previous studies on this issue differ significantly.

Several studies have evaluated the outcomes of intracytoplasmic sperm injection (ICSI) using sperm from patients with Y-chromosome microdeletions. In these studies, authors found no significant effect on embryological, clinical, or newborn baby outcomes.^[4,5]^ However, the risk of inheritance of Y-chromosomal microdeletions from father to son through ICSI is still a concern.^[6]^ Furthermore, critical questions remain as to whether the offspring of men with Y-chromosome microdeletions who underwent ICSI treatment carry any heightened risk of birth defect.

In this report, we identified a man with Y-chromosome AZFc microdeletions, who had a child through ICSI treatment without preimplantation genetic diagnosis (PGD). The newborn girl had the same abnormal chromosome inv (9) (p11q13) as her father.

## 2 Methods

### 2.1 Karyotype analysis

Peripheral blood (0.5 ml) was collected in sterile tubes containing 30 U/ml heparin. Peripheral blood lymphocytes were cultured at 37°C for 72 h in lymphocyte-culture solution (Yishengjun, Baidi Biotech Co., Ltd., Guangzhou, China), and then treated with 20 mg/ml colcemid (Sigma, UK) for 1 h. G-banding of metaphase chromosomes was performed by Giemsa staining. The numbers of chromosomes in 30 metaphase mitotic figures were counted. Karyotypes of 10 cells in mitosis metaphase were analyzed and described according to the international system for chromosome nomenclature (ISCN).

### 2.2 Detection of AZF microdeletions

Genomic DNA was extracted from peripheral blood using a commercially available whole-blood DNA extraction kit (TIAN amp Blood DNA kit; Beijing Tiangen Biotech, China). Control DNA samples obtained from unrelated males with proven fertility, and from females, were used as positive and negative controls, respectively. AZF microdeletion analysis was performed using multiplex polymerase chain reaction (PCR). On the basis of the recommendations of the European Academy of Andrology (EAA) and the European Molecular Genetics Quality Network (EMQN), three different regions—AZFa, AZFb, and AZFc—were analyzed with 7 sequence-tagged site (STS) specific markers: sY84, sY86, sY127, sY134, sY143, sY254, and sY255. sY14 (an STS located within the sex-determining region Y gene) and ZFX/ZFY were used as internal control primers.^[7]^

### 2.3 Protocols for ovarian stimulation and ICSI procedure

The patient's wife was 26 years old at the time of the ICSI procedure. Her sexual development, medical history, and physical examination were normal. Ovarian stimulation was performed using a long luteal-phase gonadotrophin-releasing hormone (GnRH) agonist (Tryptorelin, Ferring, Germany) protocol. ICSI was performed with ejaculated motile spermatozoa after oocyte retrieval. Two embryos were transferred on day 3 (the embryos were 6–8 cells, with <30% fragmentation, derived from 2 PN). To assess the treatment outcome, serum free β-human chorionic gonadotropin (free β-HCG) was measured at day 14 post-transfer. A clinical pregnancy was confirmed by ultrasound observation of fetal cardiac activity, 30 days after embryo transfer.

### 2.4 Prenatal diagnosis

The maternal serum concentrations of free β-HCG, α-fetoprotein (AFP), and unconjugated estriol (uE3), were measured at gestational week 16.4 by commercial time-resolved fluoroimmunoassay (AutoDELFIA1235, Perkin Elmer Life and Analytical Sciences, USA). Routine cytogenetic analysis by G-banding techniques was performed on cultured amniocytes at amniocentesis.

## 3 Case report

The patient was a 27-year-old man who came to our clinic because of 2 years of primary infertility. A physical examination was performed, which revealed normal testis volume and texture. A questionnaire was completed by the patient, and known factors affecting fertility (such as smoking, excessive alcohol intake, serious systemic disease, abnormality of the external genitalia, known hereditary/familial disorders) were excluded.

Semen analysis according to World Health Organization guidelines indicated normal ejaculate volume and severe oligoasthenospermia (sperm concentration was 0.27 million/mL, and motile sperm were rare when examined under a microscope).

**Figure 1:**
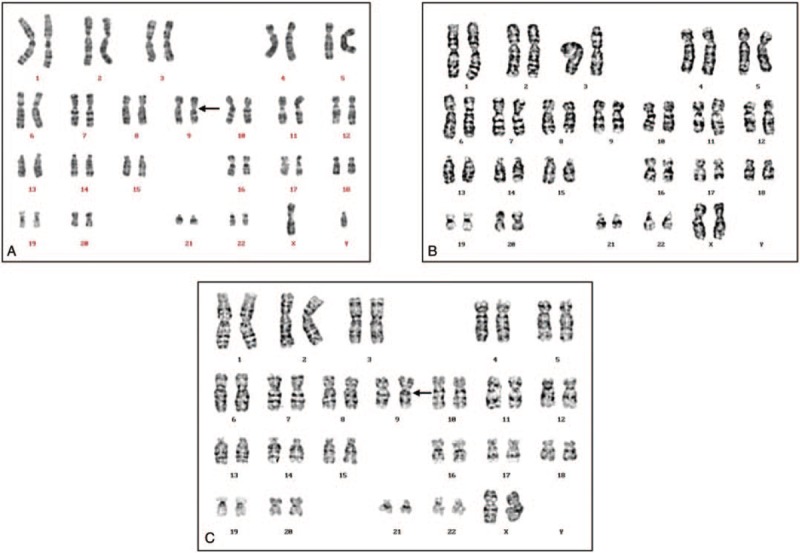
Results of karyotype analysis by G-banding. A: karyotype of the patient, from cultured peripheral blood; B: karyotype of the patient's wife, from cultured peripheral blood; C: karyotype of the baby girl, from cultured amniocytes.

**Figure 2:**
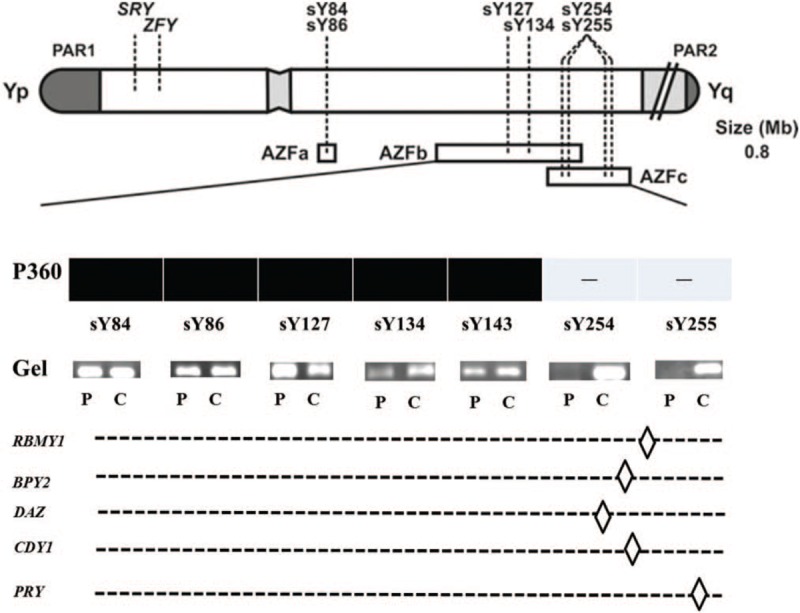
Sequence-based map of STSs on Y-chromosome microdeletions and genes with protein-coding potential. P360, patient number 360; –, absence of STSs. In gel images, P represents patient 360 and C represents a positive control. *RBMY1, BPY2, DAZ, CDY1*, and *PRY* are genes located in the AZFc subregion. AZF = azoospermia factor, STS = sequence-tagged site.

The karyotype of the patient was 46, XY, inv (9) (p11q13) (Fig. 1A). His wife's karyotype was normal (Fig. 1B). STS-based PCR analysis showed that the markers sY254 and sY255 were deleted. Other STS markers were present, including sY84 and sY86 for AZFa; and sY127, sY134, and sY143 for AZFb; indicating a lack of deletions in these regions (Fig. 2). The patient and his wife chose to undergo ICSI treatment after genetic counseling. Nine oocytes were retrieved; 2 of these were successfully fertilized. On day 3, two embryos (6IV, 8II) were transferred to the uterus of the patient's wife with endometrial level of 10A. Fourteen days after embryo transfer, the serum β-HCG concentration was 256.3 mIU/ml, confirming the pregnancy. An ultrasound scan at 7 weeks of gestation revealed a single pregnancy with cardiac activity. The prenatal ultrasound found no significant abnormality. A healthy girl was born by cesarean section at 39 weeks of gestation, weighing 3.4 kg and measuring 50 cm in length. Second-trimester maternal serum triple-screening showed that the pregnancy was high risk for trisomy 21 syndrome (risk value: 1 in 150). The karyotype of the fetus, from cultured amniocytes, was 46, XX, inv (9) (p11q13) (Fig. 1C).

This study was approved by the Ethics Committee of the First Hospital of Jilin University, and informed written consent was obtained from the patient for the publication of this case report and accompanying images.

## 4 Discussion

We report that a man with Y-chromosome AZFc microdeletions transmitted an abnormal karyotype (inv (9) (p11q13)) to his baby girl, who was conceived after ICSI. Prenatal diagnosis showed no birth defects, although the result of second-trimester maternal serum screening showed a high risk for trisomy 21 syndrome (risk value: 1 in 150).

AZFc deletions are the most frequent molecular cause of spermatogenic failure in men with nonobstructive azoospermia or severe oligozoospermia. Nonetheless, men with AZFc microdeletions and severe oligozoospermia or azoospermia are able to reproduce via assisted reproductive technologies such as ICSI (Table 1). Indeed, it has been reported that men with AZFc deletions can achieve similar rates of viable embryo production, and pregnancy, as men without AZFc deletions.^[8,9]^ Significantly, several cases have also demonstrated the transmission of AZFc deletions to offspring via natural conception.^[10,11]^ Here, the patient and his wife chose to undergo ICSI treatment, and a baby girl resulted from transplantation of the first embryo. This case is an important reminder that ICSI can be provided to men with AZFc deletions (after genetic counseling), if spermatozoa can be obtained. As such, ICSI offers hope for severely oligozoospermic or azoospermic men with AZFc deletions who wish to conceive children from their own gametes.

**Table 1:**
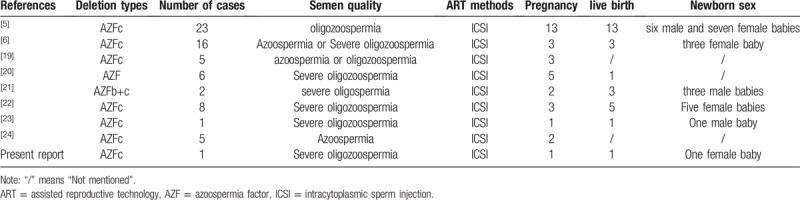
Outcomes of ART on patients with Y chromosome AZFc microdeletions.

In our study, an abnormal karyotype (inv (9) (p11q13)) was transmitted from the patient with primary infertility to his baby girl, through ICSI. Notably, the newborn with inversion 9 had no phenotypic abnormalities. To date, inv (9) (p11q13) is the most commonly observed, structurally balanced, rearrangement of chromosomes. While controversial, most cytogeneticists believe that this variant is a chromosomal polymorphism of the normal human karyotype that is without clinical significance. However, many clinical investigators have associated inv (9) with clinical diagnoses such as infertility, recurrent miscarriages, azoospermia, and congenital anomalies.^[12]^ Notably, Sasiadek et al reported inv (9) in 2.3% of all couples presenting with infertility and recurrent spontaneous abortions.^[13]^

The mechanisms by which this variant may cause infertility remain unclear and research at the molecular level will be required to understand the significance of such chromosomal breakpoints. García-Peiró et al found that male patients with infertility and inv (9) karyotype have high levels of sperm DNA fragmentation, significant meiotic alterations, anomalous aneuploidy, and altered seminogram parameters. All of these factors can result in chromosomal imbalances in progeny.^[14]^ In addition, there are numerous publications suggesting an association of inv (9) with a range of diverse clinical findings; however, long-term psycho-developmental assessment is lacking. For now, a prenatal diagnosis of inv (9) presents a decision-making dilemma for patients and clinicians. The potential outcomes of this balanced chromosomal rearrangement, and the appropriateness of options available for reproductive assistance, need further attention.

In our study, although prenatal diagnosis showed no birth defects, the result of second-trimester maternal serum screening showed a high risk for trisomy 21 syndrome (risk value: 1 in 150). With increasing numbers of children being born via ICSI procedures, greater attention has focused on the safety of ICSI. To date, most evidence demonstrates that there is no difference in the rates or types of congenital malformations arising in ICSI pregnancies, compared with standard IVF pregnancies.^[15–17]^

It has been suggested that values of prenatal screening serum markers can be influenced by ART, when screening for trisomy 21.^[18]^ Such variations in biochemical markers could have an important impact for clinical practice. Particularly in the case of assisted reproductive technology (ART) pregnancies, it can be difficult for patients to accept the appropriateness of undergoing antenatal diagnostic testing, owing to the 1% attendant risk of spontaneous miscarriage. Therefore, it would be of interest to analyze the impact of a high-risk trisomy-21 result, as to whether there is also an increased risk of the need for an invasive antenatal diagnosis. Certainly, the significance of a difference in trisomy-21 risk estimation between ART and normal pregnancies would require a multicenter study on a much larger scale.

In conclusion, we confirm that an infertile man with AZFc microdeletions can conceive his own child via ICSI technology. In such cases, high-risk results of prenatal screening with abnormal biochemical markers can be detected. In addition, the karyotype inv (9) (p11q13) has normal clinical significance and can be transmitted to offspring.

## Author contributions

**Data curation:** Linlin Li.

**Funding acquisition:** Ruizhi Liu.

**Methodology:** Xiaonan Hu, Haibo Zhu, Dongfeng Geng.

**Project administration:** Ruixue Wang.

**Supervision:** Ruizhi Liu, Ruixue Wang.

**Writing – original Draft:** Xiangyin Liu.

**Writing – review & Editing:** Cong Hu.
